# Feature Comparison and Process Optimization of Multiple Dry Etching Techniques Applied in Inner Spacer Cavity Formation of GAA NSFET

**DOI:** 10.3390/nano16020145

**Published:** 2026-01-21

**Authors:** Meng Wang, Xinlong Guo, Ziqiang Huang, Meicheng Liao, Tao Liu, Min Xu

**Affiliations:** 1 College of Integrated Circuits and Micro-Nano Electronics, Fudan University, Shanghai 200433, China; 22112020117@m.fudan.edu.cn (M.W.); xlguo22@m.fudan.edu.cn (X.G.); huangzq22@m.fudan.edu.cn (Z.H.); mcliao25@m.fudan.edu.cn (M.L.); 2 School of Microelectronics, Fudan University, Shanghai 200433, China

**Keywords:** dry etching, inner spacer cavity etching, gate-all-around nanosheet field effect transistor, SiGe selectivity etching, Si/SiGe stack structure

## Abstract

The inner spacer module, which profoundly affects the final performance of a device, is a critical component in GAA NSFET (Gate-all-around Nanosheet Field Effect Transistor) manufacturing and necessitates systematic optimization and fundamental innovation. This work aims to develop an advanced SiGe etching process with high selectivity, uniformity and low damage to achieve an ideal inner spacer structure for logic GAA NSFETs. For three distinct dry etching technologies, ICP (Inductively Coupled Plasma Technology), RPS (Remote Plasma Source) and Gas Etching, we evaluated their potential and comparative advantages for inner spacer cavity etching under the same experimental conditions. The experimental results demonstrated that Gas Etching technology possesses the uniquely high selectivity of the SiGe sacrificial layer, making it the most suitable approach for inner spacer cavity etching to reduce Si nanosheet damage. Based on the results, in the stacked structures, the SiGe/Si selectivity ratio exhibited in Gas Etching is ~9 times higher than ICP and ~2 times higher than RPS. Through systematic optimization of pre-clean conditions, temperature and chamber pressure control, we successfully achieved a remarkable performance target of cavity etching: the average SiGe/Si etching selectivity is ~56, the inner spacer shape index is 0.92 and the local etching distance variation is only 0.65 nm across different layers. These findings provide valuable guidance for equipment selection in highly selective SiGe etching and offer critical insights into key process module development for GAA NSFETs.

## 1. Introduction

Compared with traditional Fin field effect transistors (Fin FETs), GAA NSFETs implement the three-dimensional stacking idea at the transistor level. The fully surrounding gate can suppress short-channel effects, thus reducing device leakage, while vertically stacked sheets can deliver higher drive current without increasing the layout area [1,2,3,4,5]. However, such innovations bring not only performance gains but also the challenge of new processes. Because GAA NSFETs are fabricated from alternating Si/SiGe epitaxial layers for designing horizontal channels, compared to traditional processes, how accurately the etching selectivity is controlled between SiGe and Si has an important impact on the final structure and electrical performance of the transistor [6,7,8].

The inner spacer module is arguably the most critical yet challenging part of GAA NSFET manufacturing, in which the SiGe of alternating Si/SiGe layers is recessed to a precise distance and dielectric is subsequently filled to form isolation. The etching process demands an ultrahigh selectivity of SiGe to Si to avoid Si damage. Otherwise, an obvious thinning or roughening of the channel layers would degrade carrier mobility. And the quality of source/drain epitaxy would be impacted by the damaged end of the Si nanosheet, thus raising the contact resistance [9,10]. The cavity etch profile is also critical. Only an approximately rectangular profile can guarantee the uniform electrical and stress field distribution, while a “crescent-like” shape would induce the collapse in structure or dispersion in electrical performance [4,6,11,12]. Moreover, the effective gate length of the GAA NSFET is determined not only by the critical dimension (CD) of the dummy gate but also by the thickness of the inner spacer [13,14,15]. Therefore, cavity etching uniformity should also be accurately controlled. All of the above make the process very difficult.

Research on isotropic and highly selective etching of SiGe began early, and various etching methods such as wet, dry plasma and Gas Etching have been developed by researchers accordingly [16,17,18,19,20]. Wet chemistries employ strong oxidants and etchants simultaneously oxidize and dissolve SiGe in a quasi-single step [21,22]. Dry plasma etching generally adopts halogen-containing gases (such as CF_4_ or NF_3_) together with Ar, He or O_2_ to realize SiGe selective etching. Concretely, early research relied mainly on typical local plasma sources, such as ICP [23]. To minimize physical bombardment and allow chemical etching to dominate, researchers should increase the chamber pressure and reduce the bias power. However, recently, RPS etching technology has been developed, which filters out charged particles and mainly uses neutral free radicals for etching and has the greater advantage of low damage [24]. Otherwise, with the continued discovery and application of highly reactive and oxidizing gases, Gas Etching technology has emerged as an increasingly promising approach for selective etching. And it was recently found that ClF_3_ is a reactive gas with satisfactory selectivity of SiGe to Si. But there has been limited reporting on associated processing.

The GAA NSFET represents an exceptionally advanced technology and no manufacturing details have been disclosed to date. Therefore, the significance of developing key module processes should not be underestimated. The inner spacer module is regarded as the most critical part in determining the performance of the GAA NSFET, but the etching selectivity, profile and uniformity control pose a great challenge. Although numerous selective etching methods for SiGe exist, most struggle to meet the high standards required in inner spacer cavity etching of GAA NSFETs. In this work, a systematic study on SiGe selective dry etching is conducted on the basis of three etching techniques, including ICP by reactive ion etching, RPS by radical etching and Gas Etching by thermal etching. Under the same experimental conditions, we evaluated their potential and comparative advantages. Through reasonable process selection and optimization, we aim to develop an advanced SiGe etching process with high selectivity, uniformity and low damage to achieve an ideal inner spacer structure for GAA NSFETs. The findings of this work provide machine selection ideas for highly selective etching of SiGe, accurate and effective control directions for the optimization of the inner spacer cavity etching process, and important insights for the development of key process modules in GAA NSFETs.

## 2. Materials and Methods

**Figure 1:**
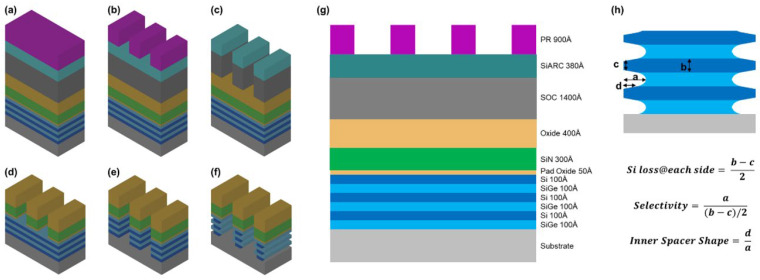
Process flow and test structure diagram to evaluate SiGe selective etching: (**a**–**f**) process flow: (**a**) film deposition; (**b**) pattern lithography; (**c**) pattern transfer to SOC; (**d**) ONO hard mask etching; (**e**) Fin etching; (**f**) cavity etching of SiGe; (**g**) specific thickness of the stacked films; (**h**) definition of evaluation criteria for the inner spacer etching morphology.

In this work, the process flow diagram shown in Figure 1 was adopted to prepare samples, and to explore the SiGe/Si selective ratios of different etching processes and optimize the process conditions of the inner spacer etching module. The 12-inch silicon wafers were cleaned before depositing the films to avoid introducing excessive impurities. The epitaxial growth of the multi-layer stacked Si/SiGe superlattice was performed by using reduced pressure chemical vapor deposition (RPCVD). The growth temperature of 600 °C was adopted, and dichlorosilane (DCS, SiH_2_Cl_2_) and germane (GeH_4_) were used as reaction precursors for Si and Ge, respectively. The pad oxide layer was grown using a thermal atomic layer deposition (ALD) method at a reaction temperature of 600 °C. Hexachlorodisilane (HCDS, Si_2_Cl_6_) and H_2_/O_2_ were used as the reaction precursor and reaction gas, respectively, which were alternately introduced to complete the reaction to control the film thickness. The SiO_2_ and SiN film layers were deposited using the plasma-enhanced chemical vapor deposition method (PECVD) with a reaction temperature of 400 °C. Among them, tetraethoxysilane (TEOS, Si(OC_2_H_5_)_4_) and O_2_ were applied to the precursor and reactive gas to grow SiO_2_, and correspondingly, silane (SiH_4_) and ammonia (NH_3_) were used to grow SiN. The spin-on carbon (SOC), silicon-containing bottom anti-reflective coating (SiARC) and photoresist (PR) were prepared through a spin-coating and baking process layer by layer. The specific stacking sequence of the film layers is shown in Figure 1a.

In this work, a deep ultraviolet (DUV) lithography machine was used for pattern exposure; the stacked layers consisting of 140 nm SOC, 38 nm SiARC and 90 nm PR were designed to be compatible with the etching process window and the anti-reflective requirements of exposure (Figure 1b). Moreover, Oxide/SiN/Oxide stacked films were designed for hard mask (ONO HM). And Si/SiGe stacked layers, in which each layer thickness is 10 nm, the stacking layer number is 6 and the Ge concentration of SiGe is 25%, were designed to simulate the stacking channels in a GAA NSFET. The specific film thickness is shown in Figure 1g. The patterning transfer on SOC film, ONO HM and Fin etching was accomplished by an anisotropic ICP etching machine (Figure 1c–e). SiGe selective etching was completed using three different etching techniques, which were named ICP, RPS and Gas (Figure 1f). The specific process parameters are presented in Table 1.

In the stacked structure, the SiGe/Si etching selective ratio, Si damage and inner spacer shape were quantitatively evaluated by the calculation method shown in Figure 1h to more intuitively observe the differences in process performance of the three etching techniques. Among the formulae, “a” and “d” denote the maximum and minimum distance of the lateral recess in the SiGe layers, respectively, whereas “b” and “c” represent the thickness of the Si layer in the inner and outer regions. Meanwhile, polycrystalline Si and monocrystalline SiGe were deposited on unstructured bare wafers to characterize the intrinsic etching selectivity and uniformity on whole wafer of different dry etching technologies. Spectroscopic ellipsometry was used to measure the film thickness for calculating etching rates. Five data points at the same radius position were measured, and then the mean value and corresponding standard deviation were calculated. The test sample was a complete 12-inch wafer. The morphologies were observed and evaluated by scanning electron microscopy (SEM, operating voltage 5 kV) and transmission electron microscopy (TEM, operating voltage 200 kV). The surface roughness of the Si layer was measured using an NX-Wafer atomic force microscope (AFM) from Park Systems (Suwon, Republic of Korea).

## 3. Results and Discussion

**Figure 2:**
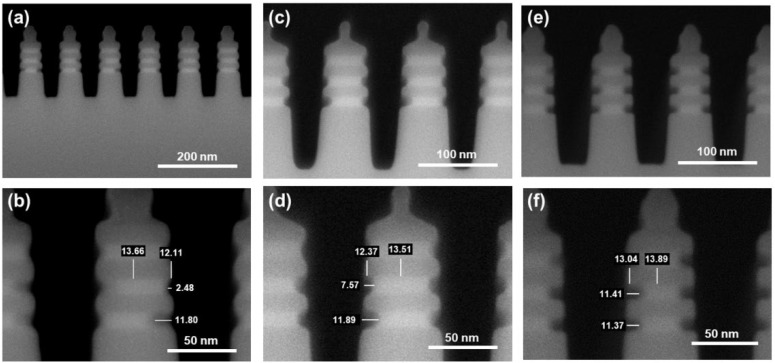
The morphological features of the inner spacer obtained by three different dry etching techniques: SEM images of (**a**,**b**) ICP etching; (**c**,**d**) RPS etching; (**e**,**f**) Gas etching.

The research content focuses mainly on the inner spacer cavity etching process. In order to better understand the differences between the three dry etching techniques of ICP, RPS, and Gas, we carefully studied the morphological differences post SiGe selective etching, as shown in Figure 2. It is worth mentioning that in each case, the same Fin etching process was adopted (Figure S1). Based on ICP technology, the smaller usable chamber pressure generates a relatively lower density of reactants, inducing the slower etching rate of SiGe in low temperature (Figure S2). Thus, we used a high temperature of 80 °C in the process to enhance the reactivity of the system. In Figure 2a,b, the obvious selective etching of SiGe is observed, but the lateral SiGe etching distances across different layers show a wide range of about 9 nm, inducing a local SiGe/Si etching selective ratio that varies in the range of 3–15. This is because ICP etching still inevitably involves the physical sputtering effect of high-energy ions even if the BRF power is set to 0. The local etching rate is significantly accelerated at the moment when the polymer passivation layer is sputtered away. This complex interaction mechanism of physical and chemical etching reduces the selective etching process window significantly, so that slight environmental drifts can trigger over-passivation or under-passivation feedback, which is detrimental to etching uniformity [17,25,26]. In addition, the end of the Si nanosheet shows a rounded morphology and has a single-sided damage of 0.78 nm, which is caused by the low etching selectivity of SiGe/Si.

Compared to ICP, RPS and Gas technologies inherently exhibit high reactivity toward SiGe. Using a high temperature led to an uncontrollable etching rate, while excessive reaction driving force resulted in a reduction in SiGe/Si etching selectivity (Figure S2). Therefore, 25 °C was selected in the experimental design. For RPS, the SiGe selective etching performance is slightly better than ICP (Figure 2c,d). The etching distance range among different regions decreases to about 4 nm, and the SiGe/Si selectivity is within the range of 13–21. The improvement in etching uniformity is mainly due to the fact that RPS technology is entirely based on the chemical etching effect of free radicals. The thickness of the passivation layer is determined by the chemical balance between deposition and volatilization, making it easier to form a uniform self-limiting surface [24]. Although the morphology of the Si nanosheet end resembles a square shape after RPS etching and the single-sided Si damage is reduced to 0.57 nm, the etching selectivity and uniformity are still not ideal. This is because the free radicals with high chemical reactivity have difficulty in making extremely high selective ratios for similar materials. In addition, the hard mask layer composed of SiO_2_ and SiN at the Fin top in RPS technology was also significantly consumed, which is the result of the *CF_x_-type free radicals themselves having a degree of reactivity to SiO_2_ and SiN. And this is disadvantageous in the actual process of the GAA NSFET. The best results are achieved using Gas Etching technology (Figure 2e,f), including almost identical etching distance in different regions, extremely high SiGe/Si etching selectivity (about 27; nine times that of ICP and twice that of RPS), completely square Si nanosheet end morphology (single-sided Si damage of 0.43 nm) and hard mask layers of SiO_2_ and SiN without obvious loss. It is worth mentioning that in order to further understand the differences among various etching techniques, apart from the etching conditions presented in Table 1, we also attempted to use the ClF_3_ + He etching body in RPS technology. Compared to the CF_4_ + O_2_ + He etching body, an uncontrolled reaction rate was observed, resulting in the over-etching condition (Figure S3). Meanwhile, the obvious damaged morphology of substrate Si also reveals the low SiGe/Si etching selectivity in RPS compared to the over-etching condition in Gas Etching using ClF_3_ + He etching body. It is worth noting that due to the etching distance having a range in local regions for ICP and RPS, the lower values were adopted when comparing their etching selectivity with that of Gas Etching. Furthermore, due to the limited resolution of SEM images which increases the measurement errors, the final process performance in our work should be benchmarked against the measurements obtained from TEM images. The essence of Gas Etching technology with excellent high selectivity lies in the significant differences in the etching activation energy of gas molecules on the different material surfaces. Due to the simplicity of the reaction process in this experimental system, there is no need to balance the etching and oxidation rate, thereby exhibiting the uniformity.

**Figure 3:**
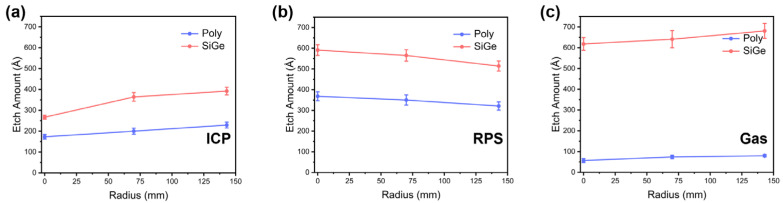
Comparison of etching rates of polycrystalline Si and monocrystalline SiGe on unstructured bare wafers using different etching machines: (**a**) ICP etching; (**b**) RPS etching; (**c**) Gas etching.

The etching rates of polycrystalline Si and monocrystalline SiGe on the whole wafer were also collected to further explore the intrinsic selectivity and uniformity of the ICP, RPS and Gas technologies, as shown in Figure 3. On this condition, the SiGe/Si selective ratios were calculated as 1.6 in ICP, 1.9 in RPS and 8.0 in Gas. Gas technology also shows the excellent selectivity throughout the whole wafer. This phenomenon is similar to that shown in structured wafers, further confirming our understanding.

**Figure 4:**
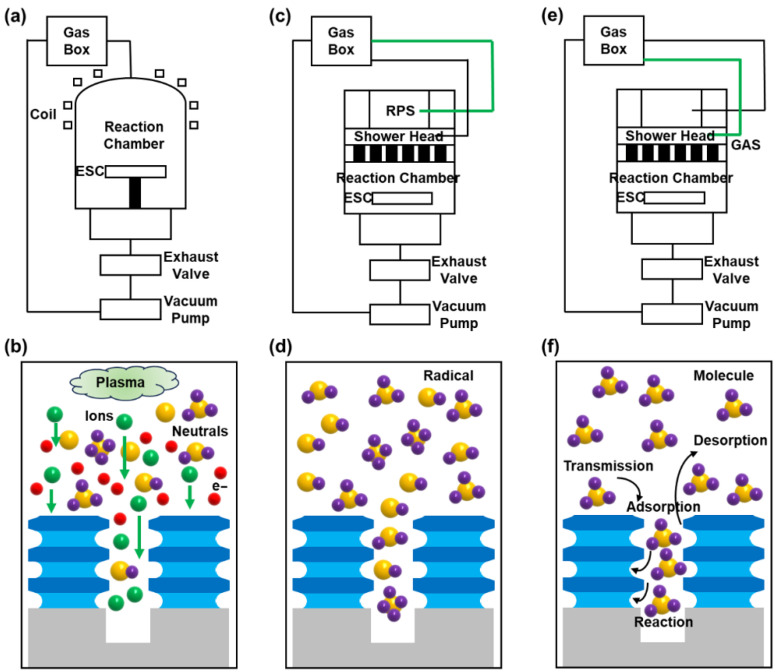
Structural diagram and working principle of the three different dry etching machines used in this work: (**a**,**b**) ICP tool; (**c**,**d**) RPS tool; (**e**,**f**) Gas tool.

A comparative analysis is conducted on the differences in the hardware structure and working principle of the ICP/RPS/Gas machines (Figure 4), to investigate the reasons why the Gas Etching technology can achieve a high selectivity etching of SiGe in Si/SiGe stacks. The ICP machine consists mainly of the following parts (Figure 4a): a coil that can produce a sharply changing magnetic field in the reaction chamber, a radio frequency power supply with a frequency of 13.56 MHz that can provide high-density plasma and a bias power supply with a frequency of 2 MHz that can control the bombardment energy and directionality of ions. The ICP tool is mainly based on plasma composed of ions, neutrals and electrons for etching (Figure 4b). Among them, although the presence of high-energy ions can rapidly bombard the wafer surface and break the chemical bonds of the material, it is difficult to achieve the high SiGe/Si selectivity etching during the inner spacer cavity etching process [25,26]. Plasma generation and etching reaction are carried out in different chambers for the RPS tool (Figure 4c), which contains only a high-frequency microwave power supply of 2.45 GHz. The reaction gas is ionized to generate plasma in the excitation chamber, and then the allowed neutral free radicals will enter the reaction chamber to react with the wafer, after the charged particles are filtered out by a specific filtering device. Charged particles do not participate during the RPS etching process, and chemical etching plays a dominant role (Figure 4d), so a relatively low-damage goal can be achieved [17,24]. However, in comparison with gas molecules, free radical etchants have a certain chemical reactivity toward most materials, which is not conducive to improving the SiGe/Si selectivity. We used the same machine as RPS to conduct the Gas Etching. But the biggest difference between them is that the Gas does not activate the plasma and directly delivers pure gas to the reaction chamber for the etching reaction (Figure 4e). Gas Etching removes atoms through the thermochemical reaction between saturated gas molecules and surface atoms, so high-energy ions or highly active free radicals will not be introduced. And the main processes include physical adsorption, chemical adsorption, surface reaction and desorption (Figure 4f). The reactivity of molecules toward materials is determined by the intrinsic reaction activation energy barriers. At specific temperatures, the activation energy barriers for etching molecules on different surfaces can vary significantly. Therefore, as long as we identify the appropriate gas molecules, there is an opportunity to achieve high selectivity in the specific reaction. In the ClF_3_ etching system, the *F transfer path is an exothermic reaction for both Si and Ge, but the activation energy of the *F transfer path from ClF_3_ to Ge is significantly lower than that to Si. Therefore, the Ge-Ge bond is more likely to break during the fluorination reaction, allowing highly selective etching of SiGe [18,27].

**Figure 5:**
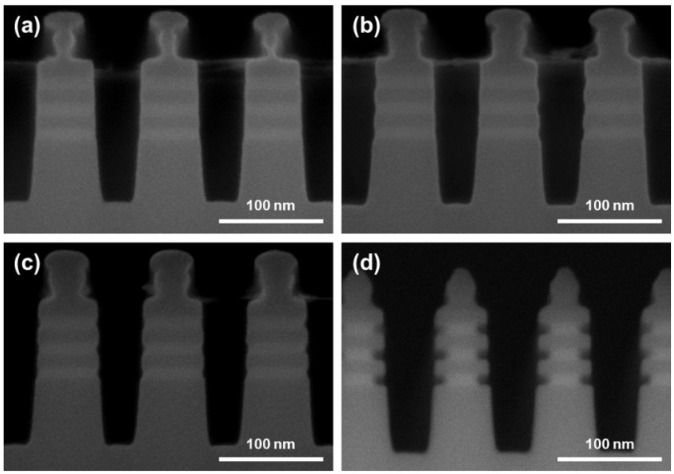
Effects of different pre-cleaning conditions on the selective removal of SiGe by dry Gas etching: (**a**) only SPM treatment; (**b**) SPM + 30 s 0.5% DHF; (**c**) SPM + 30 s 1% DHF; (**d**) SPM + 60 s 1% DHF.

Given that Gas Etching has a SiGe/Si etching selectivity higher than that of the ICP and RPS techniques, it is chosen to study the process optimization direction of the inner spacer cavity etching module. The pre-cleaning process prior to SiGe cavity etching is particularly important [28,29]. This is because an etching system that can produce heavy polymers is adopted in the Fin etching process to ensure a vertical and smooth sidewall etching morphology. An intrinsic oxide layer also exists on the SiGe or Si surface. If it cannot be thoroughly cleaned, Gas Etching will be blocked. As shown in Figure 5a, the pretreatment condition only uses SPM solution (Sulfuric/Peroxide Mixture) and no obvious SiGe or Si loss is observed during Gas Etching. On this basis, the sample is continued to be treated with a dilute hydrofluoric acid solution (DHF) with a concentration of 0.5% for 30 s after SPM cleaning. The SiGe layer is selectively etched to some extent in the subsequent Gas Etching, but the etching rate is relatively slow and the etching distance varied in different regions (Figure 5b). The SiGe etching rate does not increase significantly in the same Gas Etching process when the concentration of the DHF solution increases to 1% (Figure 5c). The high selectivity etching of SiGe and the uniform etching distance of different regions are achieved after increasing the pretreatment time of the 1% DHF solution to 60 s (Figure 5d), which shows that the intrinsic oxide layer on the Fin surface that hinders the etching of SiGe by ClF_3_ gas has been completely removed under this pre-cleaning condition. In fact, during the inner spacer etching stage of the actual preparation process flow of the GAA NSFET, the protective layer of the Fin sidewall and the dummy gate sidewall are SiO_2_ (ALD) and SiN, respectively. Thus, the consumption of the ONO HM is also explored under different pre-cleaning conditions, as shown in Figure S4.

**Figure 6:**
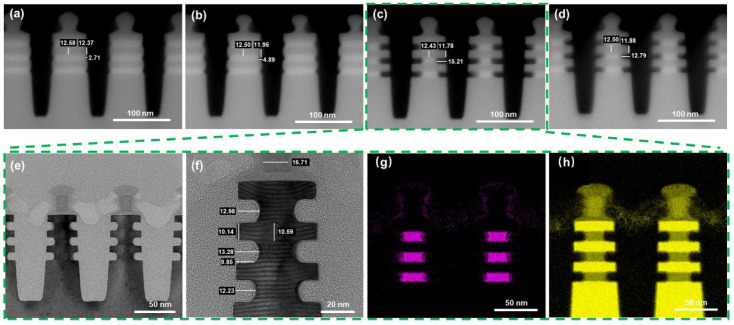
SEM and TEM images of selective etching for SiGe under different temperature conditions in the Gas etching process: (**a**) 5 °C; (**b**) 15 °C; (**c**) 25 °C; (**d**) 35 °C; (**e**–**h**) TEM and EDX mapping images of the inner spacer cavity etching at 25 °C: (**e**) low-resolution TEM image; (**f**) local enlarged TEM image; (**g**) elemental Ge EDX maps; (**h**) elemental Si EDX maps.

Temperature is considered to be one of the key parameters that influence the gas phase etching process. Higher temperatures are not conducive to the formation of stable chemical adsorption of molecules, while lower temperatures may not reach the activation energy barrier of the etching reaction [30]. Therefore, it is possible to realize the optimal etching selectivity only by finding the appropriate temperature that balances the transmission, adsorption, reaction and desorption rates. The etching rates of SiGe and Si are investigated at temperatures of 5, 15, 25 and 35 °C, as shown in Figure 6, and the corresponding etch rates of SiGe are 0.09, 0.16, 0.51 and 0.43 nm/s, respectively. With an increase in temperature, the etching rate of SiGe shows a trend that firstly increases and then decreases. From this it can be seen that the optimal temperature of the system studied in this work is 25 °C, in which a square morphology of the Si nanosheet end is also presented. Otherwise, in this reaction system, the etching rate of Si is insensitive to temperature and there is almost no loss of Si. This may be because the energy of this system is insufficient to meet the etching activation energy of Si. TEM characterization is also performed to more clearly evaluate the various indicators of inner spacer cavity etching at 25 °C (Figure 6e–h). An average SiGe/Si etch selectivity of 57.0, an inner spacer shape index of 0.74 and a SiGe etching distance range of 1.05 nm in different layers are observed. It can be observed from the EDX mapping results that there is no residue of Ge elements in the SiGe etching area or Ge enrichment on the interface, which can prove the reliability of this process.

**Figure 7:**
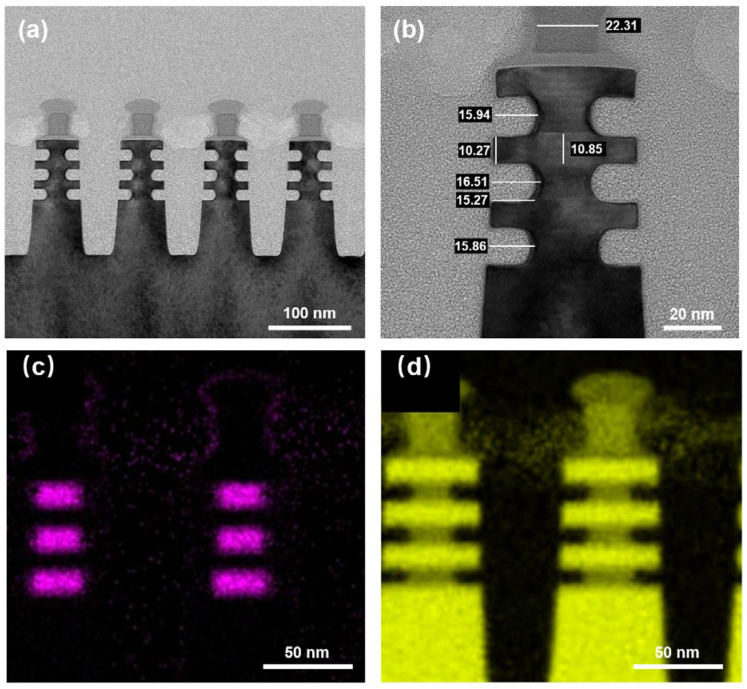
TEM and EDX mapping images of inner spacer cavity etching after chamber pressure optimization: (**a**) low-resolution TEM image; (**b**) local enlarged TEM image; (**c**) Ge EDX elemental maps; (**d**) Si EDX elemental maps.

It is often observed that the etching distance of the bottom layer is shallow in multi-layer stacked structures, especially when there are many stacked layers [31,32]. In addition, a slower etching rate is seen at the interface of the stack than at the etching center, resulting in a “Crescent-shaped” etching topography. This may be due to the difference in local etching rates caused by the restriction of reaction supplies transmission. The transmission and adsorption efficiency of the gas molecules can be improved by appropriately increasing the chamber pressure, making the etching reaction and desorption become the rate-limiting steps of the entire reaction, thereby achieving the purpose of enhancing the uniformity of etching rates in different micro-regions, as shown in Figure 7. After increasing the chamber pressure from 0.5 to 1 Torr, an average SiGe/Si etching selective ratio of 56 is obtained, which is almost consistent with the previous result (~57). However, the inner spacer shape index has improved significantly from 0.74 to 0.92. Of course, the closer this index is to 1, the better. The SiGe etching distance range of different stacked layers has also decreased from 1.05 to 0.65 nm due to the performance improvement of the bottom layer. The single-sided loss of Si is also reduced to 0.29 nm. The correctness of this process optimization strategy is proved by the above results. Based on AFM test results, the Si surface roughness has no significant changes before and post etching, which confirms the low damage nature of our process (Figure S5). In addition, the cavity etching of the inner spacer requires a certain process window to freely adjust the etching depth, thereby meeting the electrical requirements of the GAA NSFET. In this work, the relationship between the etching distance and time is also studied, as shown in Figure S6. Within the 5–60 s time range, average etching distances of 2.73, 3.64, 11.25, 19.98 and 27.72 nm are observed as the process time increases. All SiGe has been removed at 80 s, but it can still be seen that the Si nanosheet end is extremely square, with no obvious loss. It is proven that the Gas Etching process conditions optimized in this work have superior etching selectivity and a stable cavity etching process window with controllable etching depth. Meanwhile, we have also investigated the influence of both Ge concentration and thickness of SiGe layers on the Gas Etching process. For this purpose, SiGe/Si stacks were fabricated with designed variations in SiGe layer thickness (20 or 40 nm) and Ge concentration (20 or 40%). Under the same process conditions, our proposed process consistently achieves excellent SiGe/Si selectivity across different layers (Figure S7). These results demonstrate the robust versatility and practical applicability of the proposed process.

## 4. Conclusions

This study comprehensively conducts a comparative analysis of basic principles, tool hardware differences and etching performance for three SiGe selective dry etching technologies. The key challenges in controlling the inner spacer morphology, improving the SiGe/Si selective ratio, optimizing the uniformity of the etching distance and reducing the Si damage have been solved on the basis of the Gas Etching technology with the highest intrinsic selectivity. Gas Etching has been shown to have the best performance in all aspects among the other dry etching techniques. The SiGe/Si selective ratio of Gas Etching is nine and two times that of ICP and RPS etching in multi-layer stacked structures, the etching distance in different regions is almost the same and the Si damage is also the lowest. Through further process optimization, including the pre-cleaning conditions, process temperature and pressure control, the best etching results are achieved using Gas Etching technology, including a minimal etching distance range of 0.65 nm, an extremely high SiGe/Si etching selectivity of ~56 and an inner spacer shape index of 0.92. This work provides a robust optimization direction and a broad etching strategy for the inner spacer module in GAA NSFETs.

## Figures and Tables

**Table 1:** Comparison of key process parameters for SiGe selective etching using three etching techniques.

Technology	Etch Gas	SRF Power	BRF Power	Pressure	Temperature
ICP	100CF_4_ + 75O_2_ + 100He	600 W	0 W	15 mTorr	80 °C
RPS	100CF_4_ + 75O_2_ + 100He	1000 W	/	0.5 Torr	25 °C
Gas	50ClF_3_ + 100He			0.5 Torr	25 °C

## Data Availability

Data presented in this study are available on request from the corresponding authors.

## Supplementary Materials

The following supporting information can be downloaded at https://www.mdpi.com/article/10.3390/nano16020145/s1, Figure S1: Step-by-step SEM morphologies of the Fin etching process before the inner spacer cavity etching of the Si/SiGe stacked structure: (a) SOC etching step; (b) Fin HM ME etching step; (c) Fin HM OE etching step; (d) Fin trench etching step. Figure S2: Influences of process temperature in different dry technologies: (a) 25 °C of ICP; (b) 80 °C of ICP; (c) 25 °C of RPS; (d) 80 °C of RPS; (e) 25 °C of Gas Etching; (f) 80 °C of Gas Etching. Figure S3: Comparison of RPS and Gas Etching technologies under over-etch condition in ClF_3_ + He etching body: (a) RPS technology; (b) Gas Etching technology. Figure S4: Influences of different pre-cleaning conditions and Gas Etching processes on HM layer consumption: (a) after Fin etching; (b) SPM + 30 s 1% DHF; (c) SPM + 60 s 1% DHF; (d) after Gas Etching. Figure S5: The variation in Si surface roughness caused by Gas Etching process: (a) before etching AFM test result; (b) after etching AFM test result. Figure S6: The relationship between the etching distance of the inner spacer and the etching time under the Gas Etching process with excellent SiGe etching selectivity: (a) 5 s; (b) 10 s; (c) 20 s; (d) 40 s; (e) 60 s; (f) 80 s. Figure S7: The influence of the thickness and Ge concentration of the SiGe layer on the Gas Etching process: (a) TEM image; (b) EDX mapping; (c) Si and Ge concentration variation along the yellow arrow in (b).
