# Supplementary material for: Feature Comparison and Process Optimization of Multiple Dry Etching Techniques Applied in Inner Spacer Cavity Formation of GAA NSFET
Source: Nanomaterials (Basel). 2026 Jan 21;16(2):145. doi: 10.3390/nano16020145 (PMC12844779; doi:10.3390/nano16020145)
Supplement: Supplementary file 1 [file nanomaterials-16-00145-s001.zip › nanomaterials-4085332-supplementary.pdf]

Article

Feature Comparison and Process Optimization of Multiple Dry Etching Techniques Applied in Inner Spacer Cavity Formation of GAA NSFET

Meng Wang [1,2], Xinlong Guo [1], Ziqiang Huang [1], Meicheng Liao [1], Tao Liu [1,*] and Min Xu [1,2*]

[1] College of Integrated Circuits and Micro-Nano Electronics, Fudan University, Shanghai 200433, China
[2] School of Microelectronics, Fudan University, Shanghai 200433, China
* Correspondence: tliu14@fudan.edu.cn (T.L.); xu_min@fudan.edu.cn (M.X.)

Supplementary Materials

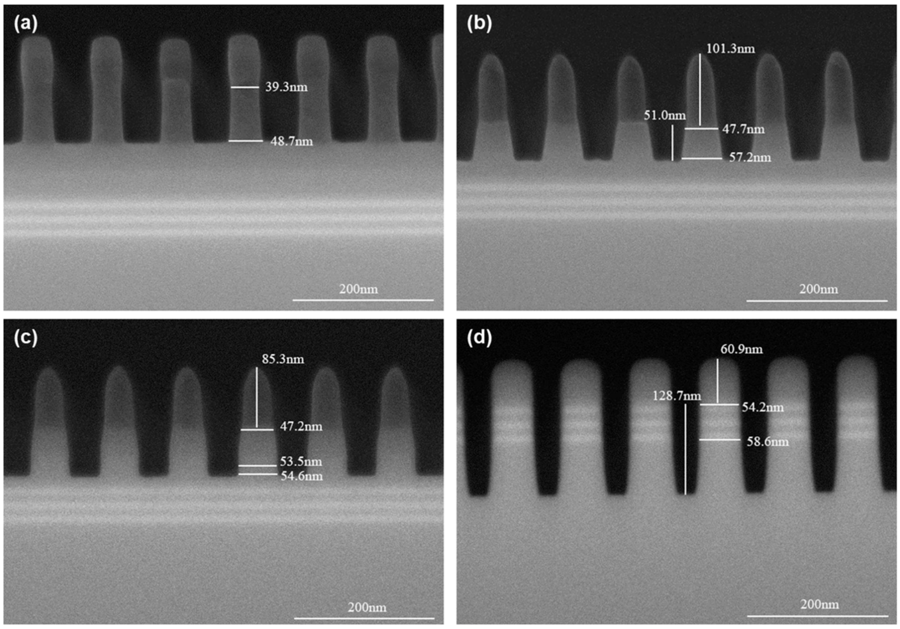

Figure S1. Step-by-step SEM morphologies of the Fin etching process before the inner spacer cavity etching of the Si/SiGe stacked structure. (a) SOC etching step; (b) Fin HM ME etching step; (c) Fin HM OE etching step; (d) Fin trench etching step.

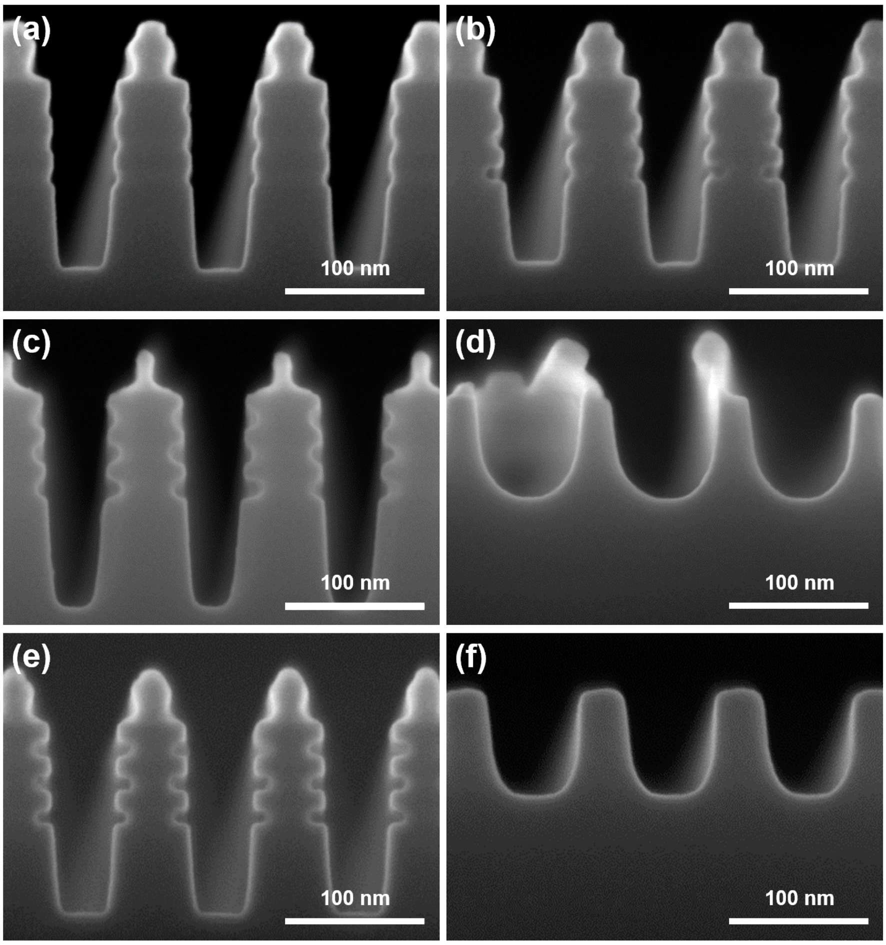

Figure S2. Influences of process temperature in different dry technologies. (a) 25 °C of ICP; (b) 80 °C of ICP; (c) 25 °C of RPS; (d) 80 °C of RPS; (e) 25 °C of Gas Etching; (f) 80 °C of Gas Etching.

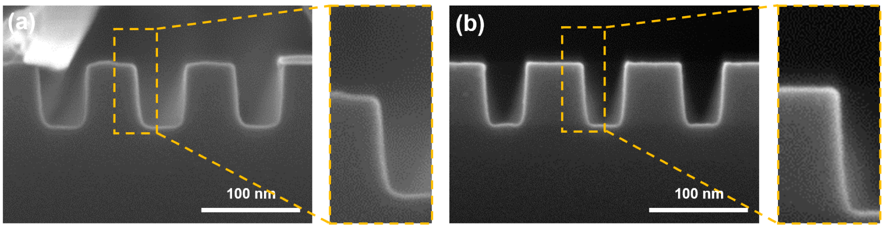

Figure S3. Compare of RPS and Gas Etching technologies under over-etch condition in ClF_3+He etching body. (a) RPS technology; (b) Gas Etching technology.

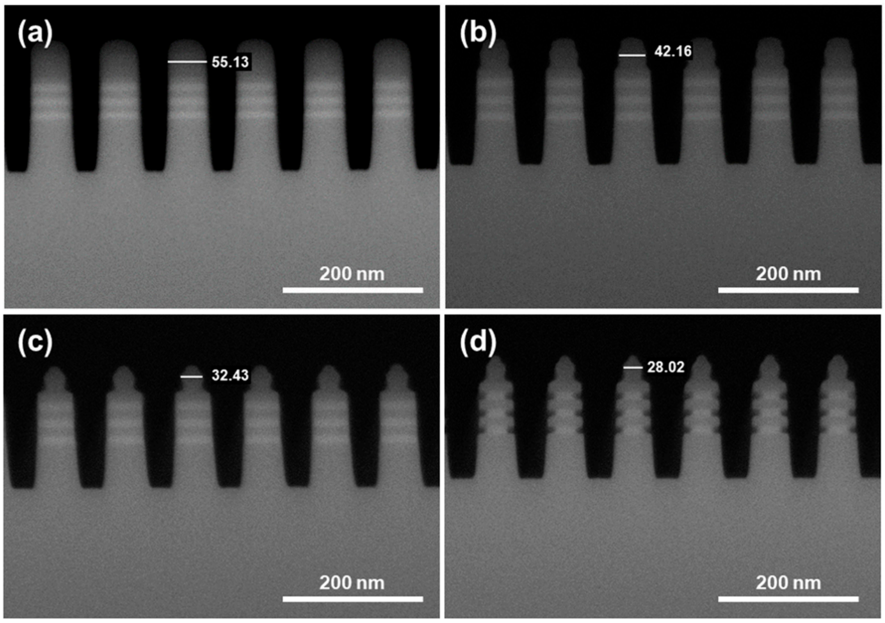

Figure S4. Influences of different pre-cleaning conditions and Gas Etching processes on HM layer consumption. (a) After Fin etching; (b) SPM + 30s 1% DHF; (c) SPM + 60s 1% DHF; (d) After Gas Etching based on the pretreatment conditions of SPM + 60s 1% DHF.

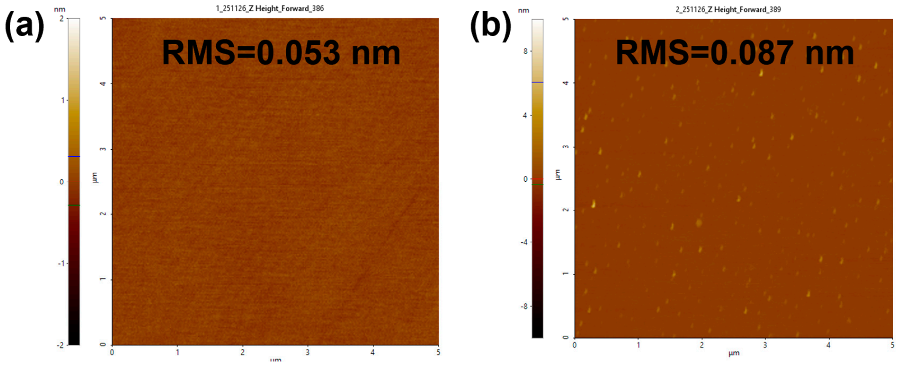

Figure S5. The variation in Si surface roughness brought by Gas Etching process. (a) before etching AFM test result; (b) after etching AFM test result.

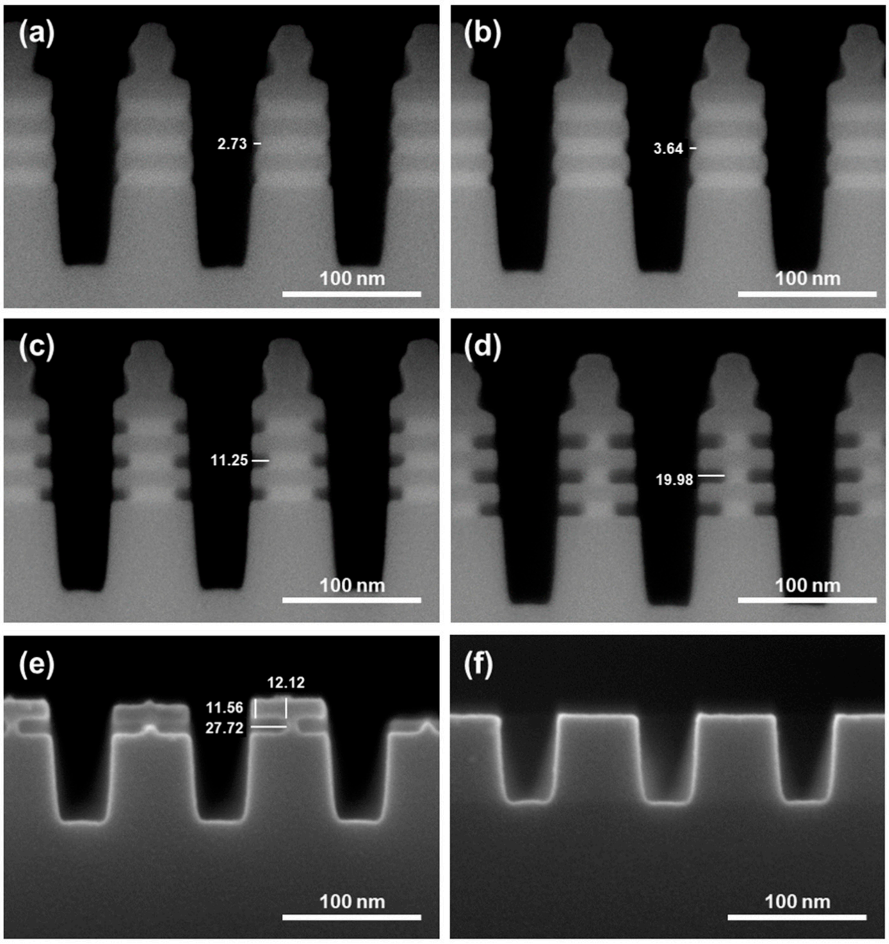

Figure S6. The relationship between the etching distance of the inner spacer and the etching time under the Gas Etching process with excellent SiGe etching selectivity. (a) 5 s; (b) 10 s; (c) 20 s; (d) 40 s; (e) 60 s; (f) 80 s.

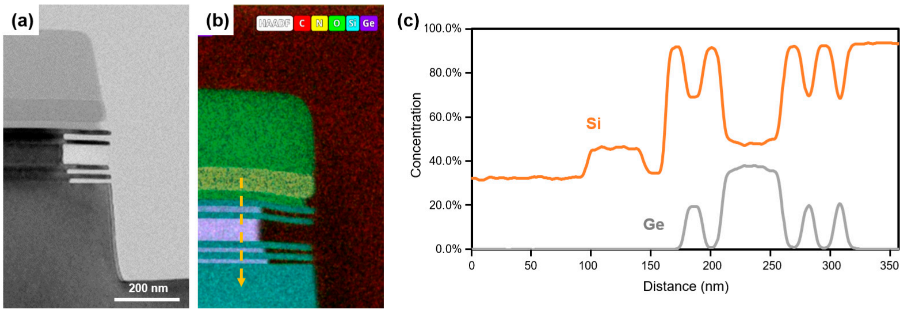

Figure S7. The influence of the thickness and Ge concentration of SiGe layer on Gas Etching process. (a) TEM image; (b)EDX mapping; (c) Si and Ge concentration variation along the yellow arrow in (b).
